# Supplementary material for: ExlA Pore-Forming Toxin: Localization at the Bacterial Membrane, Regulation of Secretion by Cyclic-Di-GMP, and Detection In Vivo
Source: Toxins (Basel). 2021 Sep 11;13(9):645. doi: 10.3390/toxins13090645 (PMC8472254; doi:10.3390/toxins13090645)
Supplement: Supplementary file 1 [file toxins-13-00645-s001.zip › toxins-1275443-supplementary.pdf]

Supplementary Materials: ExlA Pore-Forming Toxin: Localization at the Bacterial Membrane, Regulation of Secretion by Cyclic-Di-GMP, and Detection In Vivo

Vincent Deruelle, Alice Berry, Stéphanie Bouillot, Viviana Job, Antoine P. Maillard, Sylvie Elsen and Philippe Huber

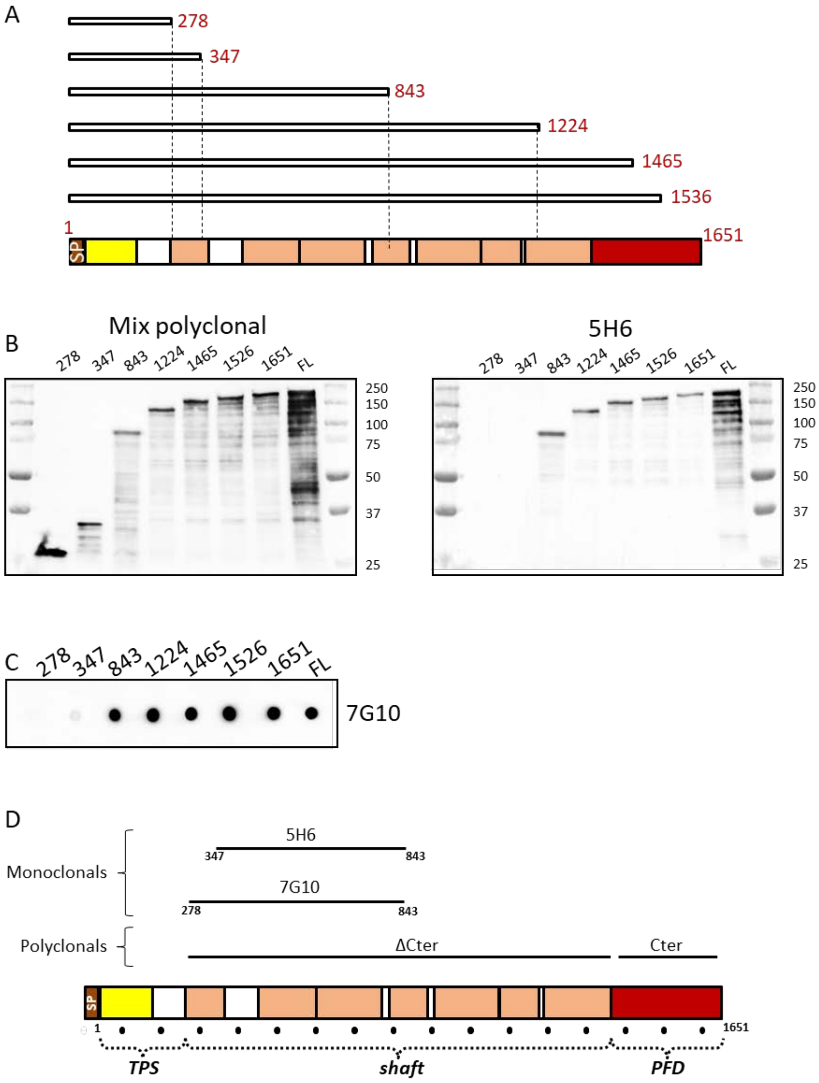

Figure S1. Antibody mapping using ExlA fragments. **A**. Diagram of the ExlA fragments used in this study. **B.** Western blot analysis of indicated fragments with either a mixture of polyclonal antibodies (Cter and ΔCter) or the 5H6 antibody. The 7G10 antibody did not yield any signals by this technique (not shown). **C.** Dot blot analysis of the same ExlA fragments with 7G10 antibody. FL, full-length protein. **D.** Summary of monoclonal and polyclonal antibody mapping using ExlA fragments. Amino-acids numbering refers to *P. aeruginosa* ExlA sequence, i.e. with the original signal peptide (SP); TPS, two-partner secretion domain, required for interaction with ExlB; shaft domain, made of type 2 filamentous-haemagglutinin repeats expected to fold as a beta-helix; PFD, pore-forming domain.
